# An enterovirus from a captive primate in China

**DOI:** 10.1186/s40064-016-2966-y

**Published:** 2016-08-08

**Authors:** Xiaochun Wang, Shihe Shao, Hua Wang, Quan Shen, Shixing Yang, Wen Zhang

**Affiliations:** Department of Pathogenic Biology, School of Medical Science, Jiangsu University, Zhenjiang, 212013 Jiangsu China

**Keywords:** Enterovirus, EV-J, Non-human primate, Phylogenetic analysis, Viral metagenomics

## Abstract

**Background:**

Enteroviruses (EVs) are a genetically and antigenically diverse group of viruses infecting humans and a variety of animals including non-human primates (NHPs). The present study was to investigate EVs in the fecal samples from captive NHPs in zoos in China using classic RT-PCR and viral metagenomics methods.

**Findings:**

An EV strain was detected in a fecal sample collected from a captive NHP of a zoo in eastern China. The complete genome of this EV strain (named Sev-nj1) was determined and characterized. Sequence analysis indicated Sev-nj1 shared the highest sequence identity (75.6 %) with an EV-J strain, Poo-1, based on the complete genome. Phylogenetic analysis showed Sev-nj1 clustered with the other EV-J strains, forming a separate clade.

**Discussion:**

According to the genetic distance-based criteria, Sev-nj1 belonged to a new type within the species EV-J. This is the first study detecting EV-J from a NHP in China, which will be helpful for the future epidemiology study of EVs in NHPs.

## Background

Enteroviruses (EVs) are members of the genus *Enterovirus* within the family *Picornaviridae* and are divided into more than 300 types to date (Van Nguyen et al. 2014). EVs infect many mammals, including humans, NHP, cattle, sheep, dog, horse, deer and pig (Van Nguyen et al. 2014; Sadeuh-Mba et al. 2014). EVs are currently classified into twelve species (labeled as EV-A—EV-H, EV-J, RhinovirusA-C) according to the sequence divergence, host range, similarities in replication and a generally observed restriction of recombination (Van Nguyen et al. 2014). Genetic identification displayed that some simian EVs isolates were similar to human viruses (e.g., EV-A, EV-B and EV-D), while others were genetically different and now divided into two separate species (e.g., EV-H and EV-J) (Van Nguyen et al. 2014; Sadeuh-Mba et al. 2014; Oberste et al. 2007, 2008, 2013a, b). Although EVs have prevalence in NHP group, whether they are associated with disease in monkeys are unclear (Van Nguyen et al. 2014; Oberste et al. 2007, 2013a, b; Nix et al. 2008).

EVs are small non-enveloped viruses having a capsid with isosahedral symmetry, whose genome is made up of a single poly-adenylated positive RNA strand with 7.5 kb in length (Larkin et al. 2007). The single open-reading frame (ORF) of EVs is flanked by two untranslated regions (5′UTR and 3′UTR), which can be translated into a polyprotein of 2200 amino acids (aa) and further processed by viral proteases into structural (VP4, VP2, VP3, and VP1) and non-structural (2A, 2B, 2C, 3A, 3B, 3C, and 3D) proteins (Piralla et al. 2013; Tang et al. 2014).

To date there are only six whole genome sequences of EV-J from samples collected from US (Oberste et al. 2002, 2007). EV-J has never been reported in China. In this study, we determined the complete genome sequence of a simian enterovirus and compared the sequence with those of EV reference strains.

## Methods

### Specimen collection

Totally, 69 fecal specimens were collected from rhesus (n = 10) and pigtailed macaques (n = 25), sooty mangabey (n = 21), and chimpanzee (n = 13), all of which are healthy and captive in three zoos in eastern China. These samples were shipped, frozen, to our laboratory and stored at −70 °C prior to analysis.

### Enterovirus identification by RT-PCR and sequencing

RNA were extraced from 200 μl of stool suspensions using the TaKaRa MiniBEST Viral RNA/DNA Extraction Kit according to the manufacturer’s instructions (TaKaRa).

Extracted RNA was tested for enterovirus (EV) by one-step Reverse transcriptase PCR (RT-PCR) assay targeting the 5′ untranslated regions as previous described (Van Nguyen et al. 2014; Oberste et al. 2003). Enterovirus complete genome was amplified by RT-nested PCR with primers designed based on the closely related EV strains (FJ007373, AF326766, AF414373 and AF414372) available in GenBank. PCR products were separated and visualized on an agarose gel, and purified by using the gel extraction kit. The resulting DNA templates were sequenced by sanger sequencing method in Sangon Sequencing company (Shanghai, China).

Viral metagenomics method was also used to identify viral sequences in these samples, the methods as previous described (Yang et al. 2016). Six separate pools were randomly generated. After low speed centrifugation and filtration the samples were treated with DNase and RNase. Six libraries were then constructed using Nextera XT DNA Sample Preparation Kit (Illumina) and sequenced using the Miseq Illumina platform with 250 bases paired ends with a distinct molecular tag for each pool.

### Phylogenetic analysis

Phylogenetic trees were constructed based on the EV**s’** VP1, 3D and complete genome nucleotide sequences in the present study and representative members of EVs. Sequence alignment was performed with the default settings in CLUSTAL W software (Larkin et al. 2007). Phylogenetic trees with 100 bootstrap resamples of the alignment data sets were established using the neighbor-joining method in MEGA.5.0 (Tamura et al. 2011). Bootstrap values (based on 1000 replicates) for each node are given. Putative ORFs in the genome were predicted by NCBI ORF finder.

### Nucleotide sequences

The sequence described here has been deposited in the GenBank database, with GenBank accession no. KT581587 and strain name Sev-nj1.

## Results and discussion

Our RT-PCR and Viral metagenomics detecting results indicated that one fecal sample from rhesus was positive for EVs. The positive rate is 1.45 % (1/69) and significantly lower than those of other research, e.g., Dung reported extremely high frequencies of active infection (95 %) among wild mandrills and other Old World monkey (Van Nguyen et al. 2014), which might be owing to the small sample number, the zoo’s clean environment and stricter monitoring system.

To investigate the relationship between Sev-nj1 and the other simian EVs, the complete genome of Sev-nj1 was determined. The genome length of the Sev-nj1 is 7298 nt. The genomes had a typical enterovirus organization, with a coding region of 2178 codons translated into four structural (VP4, VP2, VP3 and VP1) and seven non-structural proteins (2A, 2B, 2C, 3A, 3B, 3C and 3D) (Piralla et al. 2013; Tang et al. 2014).

Sequence analysis based on the whole genome indicated that Sev-nj1 showed the highest sequence similarity with strain POo-1 EV-J103, which was isolated from captive primate (Oberste et al. 2007), suggesting Sev-nj1 belongs to species of EV-J. The VP1 region of Sev-nj1 shared 67–74 % nt and 54–82 % aa identity with other strains of EV-J including EV-J108, EV-J103, SV6 and EV-J121. The other two types of EV-J, EV-J112 and EV-J115, only have 27 partial VP1 sequences (about 300 bp) (GenBank nos.: JX537988-JX538004, JX537976-JX537984, and JX538005). Based on the 300 bp sequence, Sev-nj1 shared 64–70 % sequence identities with those 27 sequences of EV-J115 and EV-J112, suggesting Sev-nj1 is genetically distant from them. Brown et al. suggested that the nucleotide identity in ‘grey zone’ of 70–75 % VP1 nt, a more stringent value of 88 % VP1 amino acid identity is more appropriate for routine typing (Brown et al. 2009). The VP1-coding sequence of Sev-nj1 showed 74 % nt and 82 % aa similarity with the EV-J103 Poo-1 strain, suggesting Sev-nj1 belongs to a new type.

**Fig. 1:**
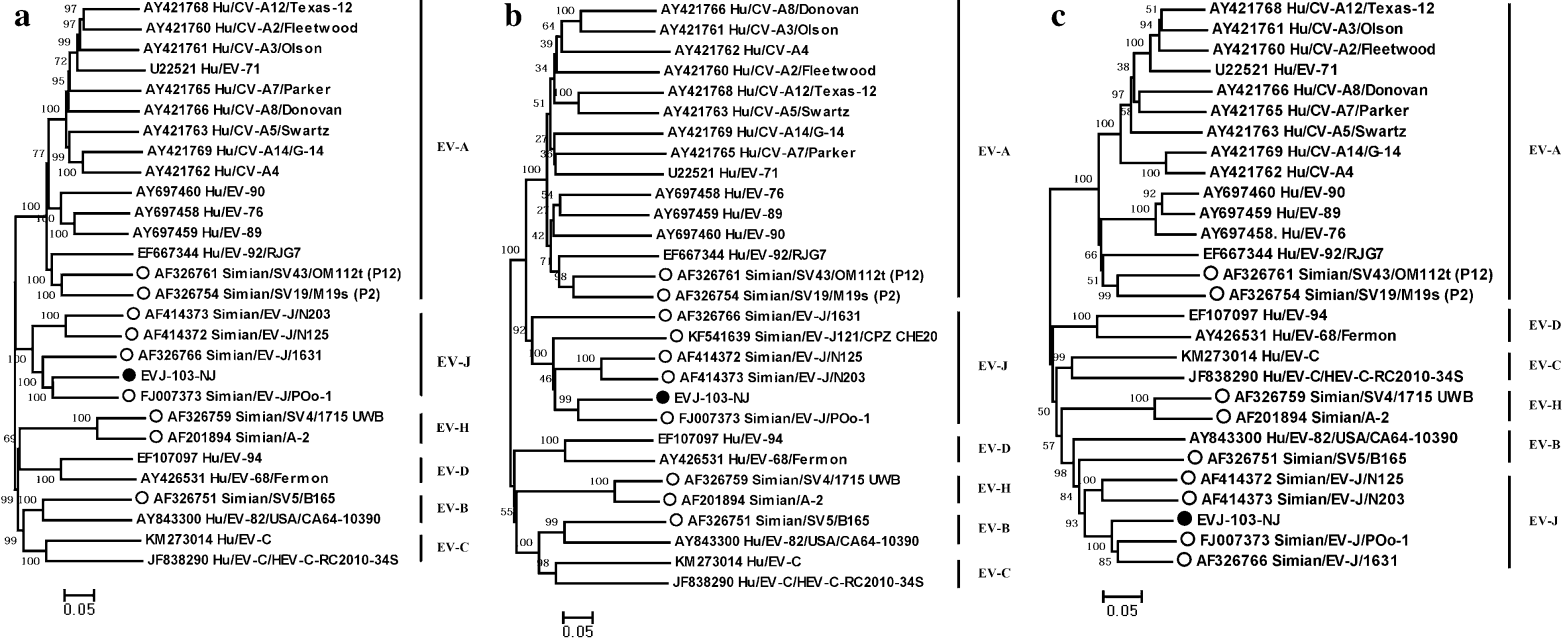
Phylogenetic analysis based on the complete genome (**a**), VP1 region (**b**) and 3D region (**c**). *Empty dots* indicate the simian EV and *black dots* indicate the strain from our study

To further elucidate the genetic relationship of Sev-nj1 with other siman EV types, phylogenetic trees were constructed based on the complete genome, VP1 and 3D regions, respectively. The virus strains in phylogenetic analysis included Sev-nj1 in the current study and the siman EV prototype strains available in the GenBank database (Fig. 1). As expected, our results indicated strain Sev-nj1 belonged to EV-J and formed a lineage with the EV-Poo-1 strain in the trees over the complete genome, VP1 and 3D regions, with a bootstrap value of 100 % (Fig. 1), which confirmed the preliminary molecular typing results. Taken together, we identified one simian enterovirus in one captive rhesus, which showed the closest relationship with a simian EV-J103 strain. Our results showed there were no detections of known human enteroviruses in NHP, indicating that human-to-primate transmission didn’t occur during the study period.

In conclusion, this is the first study identifying EV-J from a captive primate in China. Sev-nj1 is most genetically related to Ev-Poo-1, an EV-J strain isolated from pigtail macaque.
